# Evaluation of Coronavirus Disease 2019 and Influenza-like Illness Surveillance Case Definitions in a Prospective Cohort of Healthcare Personnel in Peru

**DOI:** 10.1093/cid/ciaf340

**Published:** 2025-10-06

**Authors:** Denisse Vega Ocasio, Neha N. Patel, Eva Leidman, Perrine Marcenac, Joseph Daniel Carreon, Chelsea Iwamoto, Giselle M. Soto, Matthew Westercamp, Susan Bollinger, Candice Romero, Maria Silva, Michael Prouty, Fernanda C. Lessa, Joan Neyra, Diana Ponce, Roger Castillo, Alejandro Llanos-Cuentas, Eduardo Matos, Rachel M. Smith, Ashley Fowlkes

**Affiliations:** 1 Influenza Division, National Center for Immunization and Respiratory Diseases, Centers for Disease Control and Prevention, Atlanta, Georgia, USA;; 2 Coronavirus and Other Respiratory Viruses Division, National Center for Immunization and Respiratory Diseases, Centers for Disease Control and Prevention, Atlanta, Georgia, USA;; 3 Clinical Trials Unit, U.S. Naval Medical Research Unit SOUTH (NAMRU SOUTH), Lima, Peru;; 4 Division of Healthcare Quality and Promotion, National Center for Emerging and Zoonotic Infectious Diseases (NCEZID), Centers for Disease Control and Prevention, Atlanta, Georgia, USA;; 5 Vysnova Partners, Inc. (assigned to Clinical Trials Unit, NAMRU SOUTH), Alexandria, Virginia, USA;; 6 Infectious Diseases and Tropical Medicine Department, Cayetano Heredia Hospital, Lima, Peru;; 7 Infectious Diseases Department, Arzobispo Loayza National Hospital, Lima, Peru

**Keywords:** COVID-19, influenza, surveillance, case definitions

## Abstract

**Background.:**

Public health agencies employ case definitions for influenza virus and severe acute respiratory syndrome coronavirus 2 (SARS-CoV-2) infections to optimize surveillance, monitor disease trends, and inform decision-making, particularly in limited resource settings.

**Methods.:**

Using a prospective healthcare personnel cohort in two hospitals in Lima, Peru (July 2022–June 2023), we compared and evaluated the performance of three symptom profiles of three common surveillance case definitions to identify influenza and SARS-CoV-2 infections: influenza-like illness, coronavirus disease 2019–like illness (CLI), and acute respiratory infection. Participants with an acute respiratory illness reported symptom progression twice weekly. Participants self-collected nasal swabs for SARS-CoV-2 and influenza reverse transcription real-time polymerase chain reaction testing. Sensitivity, specificity, and positive and negative predictive values of each case definition were evaluated.

**Results.:**

Of 1623 participants enrolled, 1450 illness episodes were reported; 92 (6%) were positive for influenza, 350 (24%) for SARS-CoV-2, and 3 (0.2%) were positive for both. For the four influenza-like illness case definitions evaluated, all yielded low sensitivity for detection of influenza (points estimate range, 23%–35%) and SARS-COV-2 (range, 21%–29%), but high specificity (83%–90% and 86%–92%, respectively). In contrast, the acute respiratory infection and coronavirus disease 2019–like illness definitions both yielded high sensitivity (83%–100%) and low specificity (2%–41%) for detection of both influenza and SARS-CoV-2.

**Conclusions.:**

The performance of case definitions was similar for influenza and SARS-CoV-2 supporting the use of the same definitions by surveillance networks. The substantial differences in sensitivity and specificity across the case definitions necessitate surveillance objectives and cost considerations in selecting a case definition fit for purpose.

Public health agencies rely on case definitions for respiratory illness to optimize surveillance of influenza and SARS-CoV-2 viruses. Case definitions provide standardized criteria to conduct public health surveillance based on clinical symptoms and epidemiological factors that uniquely characterize the condition of interest [1, 2]. Case definitions allow for systematic data collection that enables the comparison of disease occurrence over time, monitoring illness and virus trends, narrowing clinical diagnoses, and empowering public health officials to make informed decisions to allocate resources efficiently [1]. This standardization strengthens global health coordination, enabling timely outbreak detection and effective public health responses [2]. Updates to established case definitions are often based on available evidence used to optimize the sensitivity and specificity or to align with specific surveillance goals, such as to enhancing detections across broader age groups, incorporating new diagnostic tools, simplifying language, or adapting to an emerging pathogen [2–4].

Influenza case definitions have evolved over time, reflecting changes in understanding of the virus and the need to refine surveillance methodologies targeting seasonal variations in influenza clinical presentation [2]. Early case definitions primarily focused on clinical signs and symptoms such as fever, cough, and body aches, reflecting the observable manifestations of the illness [5]. Over time, influenza case definitions have been optimized to include clinical symptoms that are both cost-effective and easy to implement and to reflect changes in seasonal variations and meet the surveillance needs for countries, while remaining adaptable to new pathogen threats, such as severe acute respiratory syndrome coronavirus 2 (SARS-CoV-2) [3, 4].

The coronavirus disease 2019 (COVID-19) pandemic underscored the importance of adaptable case definitions as the emergence of new variants continuously required frequent updates to symptom-based screening tools. Both influenza virus and SARS-CoV-2 infections present with common, but not identical, symptoms, which has impacted the performance of existing surveillance case definitions [6]. The similarity in clinical presentation can lead to misclassification, posing a challenge for differentiating between respiratory virus infections based solely on symptoms [7, 8]. However, if the clinical presentation is sufficiently similar, existing surveillance platforms designed for influenza surveillance can be used for efficient, integrated surveillance [7, 8].

Considering the ongoing need to monitor the sensitivity and specificity of case definitions in the context of continued cocirculation of influenza and SARS-CoV-2 viruses, we used an existing prospective cohort of healthcare personnel (HCP) in Peru to evaluate the performance of three symptoms profiles for each of three surveillance case definitions to compare performance for influenza and COVID-19 illness used by the US Centers for Disease Control and Prevention (CDC), the World Health Organization (WHO), and the UK Health Security Agency. Since 2020, the US Naval Medical Research Unit SOUTH (NAMRU SOUTH) and the CDC have conducted an HCP cohort study in Lima, Peru, which presents a unique opportunity to address critical public health questions relevant for surveillance platforms used globally, as HCPs face a heightened risk of exposure to SARS-CoV-2 and influenza.

## METHODS

### Study Design and Data Collection

This study used data from a prospective cohort study of HCP conducted in two major tertiary hospitals (Cayetano Heredia Hospital and Arzobispo Loayza Hospital) in Lima, Peru, during July 2022–June 2023 [9]. HCP were eligible to participate provided they worked full-time at the facility (≥30 hours per week), had routine contact with patients, and worked at the facility for ≥1 year before enrollment. The study protocol was approved by the NAMRU SOUTH institutional review board (IRB) in compliance with all applicable federal regulations governing the protection of human subjects. The CDC IRB approved this protocol through its reliance agreement with NAMRU SOUTH. In addition, the two hospitals’ IRBs reviewed and approved the study before its implementation.

Cohort participants provided written informed consent and completed a baseline enrollment survey to report demographic characteristics, work duties, health status, and vaccination status for influenza virus and SARS-CoV-2. Participants were contacted twice per week via WhatsApp text messaging or phone calls to ask if at least one of the following key signs or symptoms were experienced within the past seven days: sore throat, cough, runny nose, body aches, fever (temperature ≥38°C), and changes in sense of taste and/or smell. An illness episode was identified if a participant experienced at least one of these key symptoms, and a nasal swab (self-collected or collected by study staff within seven days of onset of symptoms), illness onset date, and symptoms experienced were collected. Seven days after the illness onset date, participants were contacted to assess whether their illness had resolved. If the illness had not resolved, follow-up inquiries were conducted every 48 hours until resolution or up to three additional contacts (on days 9, 11, and 13 after illness onset). Within 24 hours after collection, specimens were hand delivered by participants to study staff at their facility or pickup by study staff was arranged. Specimens were transported to the NAMRU SOUTH laboratory for SARS-CoV-2 and influenza A and B viruses testing by reverse transcription quantitative real-time polymerase chain reaction [10].

### Case Definitions

We selected three different types of case definitions for respiratory disease public health surveillance, each with different symptom criteria defined by the CDC, WHO, and United Kingdom Health Security Agency. Each public health agency newly defined COVID-19–like illness (CLI) during the context of the COVID-19 pandemic and had previously established and used narrowly defined influenza-like illness (ILI) and broadly defined acute respiratory illness (ARI) (Annex 1). The selection of these case definitions as defined by the specific public health agencies for the present study were considered for this analysis because of their adaptability, comparability, and implications for public health policies and practices in both Peru and other countries. At the time of the study, the Peruvian Ministry of Health had implemented WHO’s CLI definition for the detection of SARS-CoV-2 infections and the CDC’s ILI definition for the detection of influenza infections. Comparing case definition criteria across different organizations is crucial in identifying similarities and potential inconsistencies and ensuring that public health responses are aligned and coordinated globally. It is imperative to understand these variations to harmonize surveillance data and facilitate cross-border comparisons.

### Statistical Analyses

The analysis was limited to symptomatic illness episodes with respiratory specimens that were tested for influenza and SARS-CoV-2. We conducted a descriptive analysis of influenza and SARS-CoV-2 virus detections by sociodemographic characteristics, individual symptoms, and surveillance case definitions using chi-square tests. We calculated sensitivity, specificity, positive predictive value (PPV) and negative predictive value (NPV) with 95% confidence intervals (CIs) of the 3 different surveillance case definitions for ILI, CLI, and ARI as defined by each agency, for a total of 9 comparisons against real-time polymerase chain reaction detection, as well as for individual symptoms. To evaluate the association between virus detection and individual symptoms and case definitions, we used a multivariable model to calculate adjusted odds ratios (aORs) adjusted for age, sex, and job type, accounting for repeated illness episodes. We conducted all statistical analyses in R version 4.2.2 and SAS version 9.4.

## RESULTS

### Population Characteristics

Among 1623 HCP enrolled in the cohort, 1628 illness episodes were reported. Of these, 178 (11%) illness episodes were excluded due to missing respiratory specimens. Of the remaining 1450 illness episodes, nearly half were reported among HCP aged 35 to 49 years (n = 662, 46%) with a female majority (n = 1153, 80%), having associate or bachelor’s degree (n = 1,158, 80%), and a nurse or nurse assistant (n = 844, 58%) (Table 1). While a history of influenza vaccination was reported for 1358 (94%) HCP illness episodes, only 413 (29%) were vaccinated with the 2022 influenza vaccine. Nearly all illness episodes were reported for HCP with full COVID-19 vaccination and at least 1 booster dose (n = 1440, 99%); 2 participants (0.1%) reported not completing the primary COVID-19 vaccine series.

### Influenza Virus and SARS-CoV-2 Detection

Overall, 92 (6%) illness episodes tested positive for influenza virus, 350 (24%) for SARS-CoV-2, and 3 (0.2%) were positive for both influenza virus and SARS-CoV-2 (Table 1). Detection of influenza among illness episodes varied by age, with participants aged 35 to 49 years showing higher detection rates compared to other age groups (59% and 46% respectively, *P* < .05).

### Evaluation of Individual Symptoms to Predict Influenza Virus or SARS-CoV-2 Infection

Among all illness episodes, the most reported symptoms were runny nose (85%), sneezing (75%), and sore throat (71%); however, individual symptoms varied by influenza virus and SARS-CoV-2 detection (Table 2). Influenza was associated with runny nose (aOR 4.48, 95% CI 1.35–14.88), fever (aOR 2.73, 95% CI 1.68–4.42), cough (aOR 1.92, 95% CI: 1.12–3.27), change in smell (aOR 1.80, 95% CI 1.03–3.13), and difficulty concentrating (aOR 1.71, 95% CI 1.02–2.89). Similarly, SARS-CoV-2 was associated with runny nose (aOR 3.84, 95% CI 2.21–6.66), fever (aOR 2.39, 95% CI 1.77–3.23), cough (aOR 2.88, 95% CI 2.06–4.02), and change in smell (aOR 1.79, 95% CI 1.15–2.79), and was additionally associated with body aches (aOR 2.03, 95% CI 1.52–2.71), chills (aOR 1.83, 95% CI 1.39–2.40), headache (aOR 1.56, 95% CI 1.18–2.07), sneezing (aOR 1.60, 95% CI 1.15–2.23), and diarrhea (aOR 1.46, 95% CI 1.02–2.08).

### Performance of Case Definitions to Identify Influenza Virus Infection

Overall, the performance of all ILI case definitions to identify influenza virus infections were similar with relatively low sensitivities ranging from 21% to 35%, and high specificities ranging from 83% to 91% (Table 3). The corresponding PPVs were 13% for all ILI case definitions, NPVs were 95%. Conversely, the CLI case definitions had high sensitivities ranging from 84% to 95%, but very low specificities ranging from 14% to 30% (Table 3). The corresponding PPVs ranged between 7% to 8% for all CLI case definitions, NPVs ranged from 96% to 98%. The ARI case definitions performed similarly to CLI in identifying influenza with high sensitivities and low specificities.

Influenza detection among illnesses that met any of the case definitions was 2 to 3 times greater compared to the odds of a laboratory-confirmed influenza case among those that did not meet a case definition (Table 2). The point estimates for the odds of influenza detection using ILI case definitions that required fever and cough were slightly higher than using the CLI or ARI case definitions, but all confidence intervals were overlapping.

### Performance of Case Definitions to Identify SARS-CoV-2 Infection

Similar to influenza, all ILI case definitions to identify SARS-CoV-2 infections performed similarly, with relatively low sensitivities ranging from 20% to 29%, and high specificities ranging from 86% to 93% (Table 3). Corresponding PPVs ranged from 40% to 48% for all ILI case definitions; NPVs were 79%. Conversely, the CLI case definitions had high sensitivities ranging from 85% to 93%, but very low specificities ranging from 16% to 33%. The corresponding PPVs ranged between 26% to 29% for all CLI case definitions; NPVs was 88% for all. The ARI case definitions performed similarly to CLI in identifying influenza with high sensitivities and low specificities.

Compared to patients who tested negative for SARS-CoV-2 infection, patients testing positive for SARS-CoV-2 were more likely to be captured by the WHO ILI case definition (aOR 3.66, 95% CI 2.51–5.32) and the CDC ARI case definition (aOR 3.35, 95% CI 2.39–4.70) (Table 2).

## DISCUSSION

Leveraging a prospective cohort of HCP in Peru, we evaluated the performance of global surveillance case definitions for ILI, CLI, and ARI according to the constellation of symptoms to define each by three public health agencies to detect laboratory-confirmed influenza virus and SARS-CoV-2 infections. When evaluating clinical symptoms as predictors of infection, we found that differences in symptom criteria led to minimal variation in the ability of case definitions to detect influenza and SARS-CoV-2 infections. Overall, we found that the performance of case definitions was similar in detecting influenza virus and SARS-CoV-2 infections. Although ILI case definitions exhibited higher specificity in detecting these viruses, CLI and ARI case definitions showed greater sensitivity. The comparable performance of these definitions suggests their potential suitability for use in integrated surveillance networks.

Overall, for both viruses, the ILI case definitions had lower sensitivity but high specificity, whereas the CLI and ARI case definitions had higher sensitivities but low specificity. These findings underscore the importance of considering surveillance objectives in selecting case definitions. Our findings are consistent with other studies that have observed high specificities and lower sensitivities for the detection of infection by influenza or SARS-CoV-2 viruses using the ILI definitions [8, 11, 12]. Definitions having a higher specificity are suitable in routine surveillance where the primary goal is to predict infection or monitor changes in disease prevalence over time by influenza viruses or SARS-CoV-2, requiring collection of fewer specimens to identify cases. This is important for managing resources effectively, particularly in settings where testing capacity or healthcare resources are limited. However, these definitions, because of their low sensitivity, may not capture the complete picture of infection rates as they miss a significant proportion of influenza virus and SARS-CoV-2 infections [2, 8, 11, 12].

In our study, the ARI case definitions that only required the presentation of a single symptom from a symptom array captured nearly all cases, resulting in a very low specificity. Conversely, we observed that adding fever in the case definition, as seen in the CDC definition, led to lower sensitivity and higher specificity compared to other definitions. Several studies have evaluated the impact of incorporating fever as a criterion in ARI case definitions, with results indicating that fever-inclusive definitions may overlook a substantial number of true cases of influenza and SARS-CoV-2 cases [13–15]. Using a broader definition can optimize detection when surveillance objectives are focused on complete case capture, which is often necessary for HCP who may present with varied symptoms [2, 16]. This is crucial in a high-risk population where early detection and isolation can prevent further transmission; however, it may increase test volume and therefore cost associated with surveillance [16]. The single-symptom ARI definitions may similarly be important for use in outbreak settings when capturing every case is critical [2]. Recognizing that the relative tradeoff between sensitivity and specificity of case definitions varies by the intended use of the surveillance system, it is crucial to consider these limitations when defining surveillance goals [2, 11, 17].

Reports of runny nose, cough, and fever were significantly associated with testing positive for both influenza virus and SARS-CoV-2 infections, whereas body aches and chills were significantly associated with testing positive only for SARS-CoV-2 infections. Cough has been the most commonly associated symptom with influenza detection and has consistently been part of the WHO ILI case definition since 1999 [18–20]. Runny nose has also been found to be significantly associated with influenza virus and SARS-CoV-2 infection [21–23] but is far too vague to have much utility. Similarly, a study conducted in 2023 found that the symptoms associated with SARS-CoV-2 infection can vary according to the SARS-CoV-2 strains, with the emergence of the Omicron variant being linked to an increase in coughing, sore throat, runny nose, and sneezing [24]. The variability of symptoms for SARS-CoV-2 infection underscores the importance of pairing symptom-based surveillance with virologic testing [25].

### Study Limitations

This study is subject to several limitations. First, the limited number of influenza detections may have yielded lower positive predictive values, which decrease during periods of lower disease prevalence. Although our NPV and PPV estimates may be reduced, case definition sensitivity and specificity are less vulnerable. Second, because the HCP population represent working-age adults, we were not able to evaluate the case definitions among pediatric populations or older groups; thus, our estimated rates of respiratory infections might not be generalizable to other age groups. Third, this study relied on self-reported symptoms, which may introduce bias from recall inaccuracies, underreporting of mild symptoms, or misclassification of symptoms onset; future research should incorporate objective diagnostic measures to enhance data reliability. Replicating this analysis among other age groups would be useful in evaluating the applicability of our findings, particularly as other studies have noted differences in presentation among children and older adults [26].

## CONCLUSION

Using a longitudinal cohort of HCP, we found similar performance of different criteria used in published case definitions of ARI, CLI, and ILI for the detection influenza virus and SARS-CoV-2 infections. Although ILI case definitions were more specific to detect influenza virus and SARS-CoV-2 infections, CLI and ARI case definitions were more sensitive, and the current similarities in performance suggest that these definitions may be used in the context of integrated surveillance networks. We also observed that for a given case definition, the performance is comparable for both pathogens, suggesting that a single case definition could be effectively utilized for both. However, in several countries, distinct case definitions are currently employed for influenza and SARS-CoV-2 detection. Our data indicate that adopting a unified case definition within a given country could streamline data collection processes. Moreover, due to the substantial overlap in symptomatology of these viruses, our study underscores the importance of regularly reviewing and refining symptom-based screening tools to enhance early detection and maximize efficient use of resources. By doing so, we can improve the accuracy and efficiency of testing, ultimately leading to better outcomes for patients and HCP alike.

## Figures and Tables

**Annex 1:** Symptom profiles for respiratory virus surveillance case definitions as used by public health agencies.

	Case Definitions		
	Influenza-Like-Illness (ILI)	COVID-19-Like-Illness (CLI)	Acute Respiratory Illness (ARI)
Public Health Agency			
Centers for Disease Control and Prevention (US CDC)	Fever accompanied by either a cough or sore throat or both [27]	Acute onset or worsening of at least 2 of the following symptoms or signs: fever, chills, myalgia (body ache), headache, sore throat, nausea or vomiting, diarrhea, fatigue, congestion, or runny nose [28].	At least 2 of the following: fever, cough, runny nose, or nasal congestion [27]
World Health Organization (WHO)	Fever accompanied by cough with onset within the last 10 d [25].	Presence of at least 3 of the following: fever, cough, general weakness/fatigue, headache, myalgia, sore throat, coryza, dyspnea, nausea/diarrhea/anorexia [29].	At least 1 of the following: cough, sore throat, shortness of breath, or coryza [30]
United Kingdom Health Security Agency (UK HSA)	Fever accompanied by cough [31]	Presence of at least 1 of the following: fever, cough, change in smell, change in taste [32]	Sudden onset of symptoms and at least 1 of the following: cough, sore throat, shortness of breath, or coryza (nasal symptoms such as congestion or discharge) [31]

**Table 1.** Characteristics of Healthcare Personnel With at Least 1 Respiratory Symptom, Peru, 2022–2023

	All Symptomatic Illness Episodes		Influenza Positive Versus All Episodes			SARS-CoV-2 Positive Versus All Episodes		
Characteristic	No.	%	No.	%	*P*	No.	%	*P*
Total	1450	100%	92	100%	–	350	100%	–
Age (y)								
20–34	419	28.9%	24	26.1%	.01	92	26.3%	.24
35–49	662	45.7%	54	58.7%		155	44.3%	
50–64	323	22.3%	10	10.9%		91	26.0%	
65+	46	3.2%	4	4.3%		12	3.4%	
Sex								
Male	297	20.5%	19	20.7%	1.00	74	21.1%	.78
Female	1153	79.5%	73	79.3%		276	78.9%	
Race/ethnicity								
Mixed	1293	89.2%	82	89.1%	.75	309	88.3%	.57
Indigenous	119	8.2%	9	9.8%		32	9.1%	
Black/Afro-Peruvian	13	0.9%	1	1.1%		2	0.6%	
White	17	1.2%	0	0.0%		3	0.9%	
Other	4	0.3%	0	0.0%		2	0.6%	
Missing	4	0.3%	0	0.0%		2	0.6%	
Highest education level								
Less than a high school or no degree	83	5.7%	7	7.6%	.38	18	5.1%	.89
Highschool	96	6.6%	4	4.3%		23	6.6%	
Associate or bachelor’s degree	1158	79.9%	77	83.7%		284	81.1%	
Postgraduate education	113	7.8%	4	4.3%		25	7.1%	
Job type								
Physician	76	5.2%	5	5.4%	.27	24	6.9%	.36
Nurse or nurse assistant	844	58.2%	56	60.9%		213	60.9%	
Technician^a^	60	4.1%	0	0.0%		15	4.3%	
Administrative staff	197	13.6%	13	14.1%		48	13.7%	
Other health professionals	104	7.2%	6	6.5%		19	5.4%	
Missing	169	11.7%	12	13.0%		31	8.9%	
Work in intensive care unit								
Yes	196	13.5%	10	10.9%	.53	48	13.7%	.93
No	1254	86.5%	82	89.1%		302	86.3%	
Work in emergency department								
Yes	417	28.8%	27	29.3%	.99	102	29.1%	.91
No	1033	71.2%	65	70.7%		248	70.9%	
Work > 40 h per wk								
Yes	127	8.8%	8	8.7%	.95	28	8.0%	.36
No	1323	91.2%	84	91.3%		322	92.0%	
Any underlying medical condition								
Yes	188	13.0%	8	8.7%	.27	46	13.1%	.98
No	1262	87.0%	84	91.3%		304	86.9%	
Influenza vaccine history								
Yes	1358	93.7%	88	95.7%	.77	322	92.0%	.15
No	60	4.1%	4	4.3%		20	5.7%	
Don’t know	15	1.0%	0	0.0%		5	1.4%	
Missing	17	1.2%	0	0.0%		3	0.9%	
Received 2022 influenza vaccine	413	28.5%	17	18%	.88	87	25%	<.001
COVID-19 Doses								
Fully vaccinated only	8	0.6%	1	1.1%	.45	1	0.3%	.79
Fully vaccinated + booster	359	24.8%	22	23.9%		86	24.6%	
Fully vaccinated + 2 boosters	1062	73.2%	67	72.8%		260	74.3%	
Fully vaccinated + >2 boosters	19	1.3%	2	2.2%		3	0.9%	
Unknown	2	0.1%	0	0%		0	0.0%	

Abbreviations: COVID-19, coronavirus disease 2019; SARS-CoV-2, severe acute respiratory syndrome coronavirus 2.

a Technician includes phlebotomist, dental technician, and laboratory technicians.

**Table 2.** Evaluating Symptoms and Case Definitions as Predictors of Influenza and SARS-CoV-2 Virus Infections in a Healthcare Personnel Cohort Study, Peru, 2022–2023

	Overall N = 1450	Influenza Detections n = 92			SARS-CoV-2 Detections n = 350		
	No. (%)	No. (%)	aOR (95% CI)^a^	*P*-value	No. (%)	aOR (95% CI)^a^	*P*-value
Symptoms							
Runny nose	1236 (85.2%)	89 (96.7%)	4.48 (1.4–14.9)	.015	335 (95.7%)	3.84 (2.2–6.7)	<.001
Sneezing	1082 (74.6%)	77 (83.7%)	1.67 (.9–3.0)	.095	285 (81.4%)	1.60 (1.2–2.2)	.005
Sore throat	1029 (71.0%)	64 (69.6%)	0.84 (.5–1.4)	.492	258 (73.7%)	1.10(.8–1.5)	.525
Cough	946 (65.2%)	72 (78.3%)	1.92 (1.1–3.3)	.017	282 (80.6%)	2.88 (2.1–4.0)	<.001
Headache	884 (61.0%)	62 (67.4%)	1.32 (.8–2.1)	.258	246 (70.3%)	1.56 (1.2–2.1)	.002
Body aches	877 (60.5%)	65 (70.7%)	1.51 (.9–2.5)	.091	257 (73.4%)	2.03 (1.5–2.7)	<.001
Chills	414 (28.6%)	31 (33.7%)	1.35 (.8–2.2)	.234	134 (38.3%)	1.83 (1.4–2.4)	<.001
Fatigue	388 (26.8%)	30 (32.6%)	1.13 (.7–1.9)	.635	114 (32.6%)	1.25 (.9–1.7)	.171
Difficulty concentrating	294 (20.3%)	30 (32.6%)	1.71 (1.0–2.9)	.044	67 (19.1%)	0.89 (.6–1.2)	.482
Earache	288 (19.9%)	24 (26.1%)	1.24 (.7–2.1)	.443	80 (22.9%)	1.13 (.8–1.6)	.464
Fever	285 (19.7%)	34 (37.0%)	2.73 (1.7–4.4)	<.00	110 (31.4%)	2.39 (1.8–3.2)	<.001
Shortness of breath	236 (16.3%)	22 (23.9%)	1.70 (1.0–3.0)	.068	64 (18.3%)	1.03 (.7–1.5)	.876
Diarrhea	206 (14.2%)	13 (14.1%)	0.75 (.4–1.6)	.448	62 (17.7%)	1.46 (1.0–2.1)	.039
Change in taste	173 (11.9%)	17 (18.5%)	1.80 (1.0–3.1)	.039	59 (16.9%)	1.32 (.8–2.1)	.244
Nausea	170 (11.7%)	18 (19.6%)	1.86 (1.0–3.4)	.040	43 (12.3%)	1.13 (.8–1.7)	.557
Change in smell	133 (9.2%)	12 (13.0%)	1.75 (.9–3.3)	.076	53 (15.1%)	1.79 (1.2–2.8)	.010
Wheezing	111 (7.7%)	11 (12.0%)	1.59 (.8–3.4)	.225	21 (6.0%)	.58 (0.3–1.1)	.076
Painful breath	99 (6.8%)	11 (12.0%)	1.46 (.6–3.4)	.381	18 (5.1%)	0.51 (.3–1.0)	.065
Vomiting	44 (3.0%)	4 (4.3%)	1.36 (0.4–4.6)	.614	13 (3.7%)	1.26 (.6–2.6)	.538
Case definitions							
Influenza-like illness							
US CDC	257 (17.7%)	32 (34.8%)	2.78 (1.7–4.5)	<.001	103 (29.4%)	2.54 (1.9–3.5)	<.001
WHO	148 (10.2%)	19 (20.7%)	2.82 (1.6–5.1)	.001	71 (20.3%)	3.66 (2.5–5.3)	<.001
UK HSA	229 (15.8%)	30 (32.6%)	2.87 (1.7–4.7)	<.001	98 (28.0%)	2.81 (2.1–3.9)	<.001
COVID-like illness							
US CDC	1251 (86.3%)	87 (94.6%)	2.46 (1.0–6.3)	.059	326 (93.1%)	2.10 (1.4–3.3)	.001
WHO	1114 (76.8%)	81 (88.0%)	2.09 (1.1–4.1)	.033	308 (88.0%)	2.36 (1.6–3.4)	<.001
UK HSA	1033 (71.2%)	77 (83.7%)	2.17 (1.2–4.0)	.012	298 (85.1%)	2.71 (1.9–3.9)	<.001
Acute respiratory illness^b^							
US CDC	936 (64.6%)	76 (82.6%)	2.68 (1.5–4.9)	.001	291 (83.1%)	3.35 (2.4–4.7)	<.001
WHO	1421 (98.0%)	92 (100.0%)	-	-	350 (100.0%)	-	-
UK HSA	1421 (98.0%)	92 (100.0%)	-	-	350 (100.0%)	-	-

Abbreviations: aOR, adjusted odds ratio; CI, confidence interval; CDC, Centers for Disease Control and Prevention; COVID, coronavirus disease 2019; SARS-CoV-2, severe acute respiratory syndrome coronavirus 2; UK HSA, United Kingdom Health Services Agency; WHO, World Health Organization.

a Adjusted odds ratio (aOR) for the odds of testing positive for influenza or SARS-CoV-2 using persons without the symptom or case as the referent group and adjusting for age, sex, and job type.

b 100% of cases met case definition therefore the odds ratio could not be calculated.

**Table 3.** Performance of Respiratory Surveillance Case Definitions for the Detection of Influenza and SARS-CoV-2 Viruses in a Healthcare Personnel Cohort Study, Peru, 2022–2023

	Influenza				SARS-CoV-2			
Case Definitions by Public Health Agency	Sensitivity % (95% CI)	Specificity % (95% CI)	Positive Predictive Value % (95% CI)	Negative Predictive Value % (95% CI)	Sensitivity % (95% CI)	Specificity % (95% CI)	Positive Predictive Value % (95% CI)	Negative Predictive Value % (95% CI)
Influenza-like illness								
CDC	34.8 (25.1–45.4)	83.4 (81.3–85.4)	12.5 (8.7–17.1)	95.0 (93.6–96.1)	29.4 (24.7–34.5)	86.0 (83.8–88.0)	40.1 (34.0–46.3)	79.3 (76.9–81.6)
WHO	22.8 (14.7–32.8)	90.0 (88.3–91.5)	13.4 (8.5–19.7)	94.5 (93.1–95.7)	20.6 (16.5–25.2)	92.3 (90.5–93.8)	45.9 (37.9–54.0)	78.5 (76.2–80.7)
UK HSA	32.6 (23.2–43.2)	85.3 (83.4–87.2)	13.1 (9.0–18.2)	94.9 (93.5–96.1)	28.0 (23.4–33.0)	88.1 (86.0–89.9)	42.8 (36.3–49.5)	79.4 (77.0–81.6)
COVID-like illness								
CDC	94.6 (87.8–98.2)	14.3 (12.5–16.3)	7.0 (5.6–8.5)	97.5 (94.2–99.2)	93.1 (90.0–95.6)	15.9 (13.8–18.2)	26.1 (23.6–28.6)	87.9 (82.6–92.1)
WHO	88.0 (79.6–93.9)	23.9 (21.7–26.3)	7.3 (5.8–9.0)	96.7 (94.2–98.4)	88.0 (84.1–91.2)	26.7 (24.1–29.4)	27.6 (25.0–30.4)	87.5 (83.5–90.8)
UK HSA	83.7 (74.5–90.6)	29.6 (27.2–32.1)	7.5 (5.9–9.2)	96.4 (94.1–98.0)	85.1 (81.0–88.7)	33.2 (30.4–36.1)	28.8 (26.1–31.7)	87.5 (84.0–90.5)
Acute respiratory illness^a^								
CDC	82.6 (73.3–89.7)	36.7 (34.1–39.3)	8.1 (6.5–10.1)	96.9 (95.0–98.2)	83.1 (78.8–86.9)	41.4 (38.4–44.3)	31.1 (28.1–34.2)	88.5 (85.4–91.1)
WHO	100 (96.1–100)	2.1 (1.4–3.1)	6.5 (5.3–7.9)	100 (88.1–100)	100 (99.0–100)	2.6 (1.8–3.8)	24.6 (22.4–27.0)	100 (88.1–100)
UK HSA	100 (96.1–100)	2.1 (1.4–3.1)	6.5 (5.3–7.9)	100 (88.1–100)	100 (99.0–100)	2.6 (1.8–3.8)	24.6 (22.4–27.0)	100 (88.1–100)

Abbreviations: aOR, adjusted odds ratio; CI, confidence interval; CDC, Centers for Disease Control and Prevention; COVID, coronavirus disease 2019; SARS-CoV-2, severe acute respiratory syndrome coronavirus 2; UK HSA, United Kingdom Health Services Agency; WHO, World Health Organization.

a The acute respiratory illness case definitions from WHO and UK HSA were similar and yield the same result values.

## Data Availability

The datasets analyzed in this study are not publicly available but are available from the corresponding author on reasonable request.

## References

[1] Centers for Disease Control and Prevention. Surveillance case definitions for current and historical conditions. National Notifiable Diseases Surveillance System (NNDSS). Available at: https://ndc.services.cdc.gov/. Accessed 19 May 2025.

[2] Fitzner J, Qasmieh S, Mounts AW, Revision of clinical case definitions: influenza-like illness and severe acute respiratory infection. Bull World Health Organ 2018; 96:122–8. 29403115 10.2471/BLT.17.194514 PMC5791775

[3] Penttinen P, Pebody R. Influenza case definitions—optimising sensitivity and specificity. Euro Surveill 2015; 20:21148. 26062644 10.2807/1560-7917.es2015.20.22.21148

[4] Groves HE, Piché-Renaud P-P, Peci A, The impact of the COVID-19 pandemic on influenza, respiratory syncytial virus, and other seasonal respiratory virus circulation in Canada: a population-based study. Lancet Reg Health Am 2021; 1:100015. 34386788 10.1016/j.lana.2021.100015 PMC8285668

[5] World Health Organization. WHO recommended surveillance standards. Geneva, Switzerland: World Health Organization, 1999.

[6] Dabaja-Younis H, Fuchs E, Shorbaji N, SARS-CoV-2 and seasonal influenza: similarities and disparities. Arch Virol 2022; 167:2761–5. 36269417 10.1007/s00705-022-05615-3 PMC9589861

[7] World Health Organization. Regional Office for Europe, WHO Regional Office for Europe guidance for influenza surveillance in humans. Copenhagen, Denmark: WHO Regional Office for Europe, 2009.

[8] World Health Organization. End-to-end integration of SARS-CoV-2 and influenza sentinel surveillance: revised interim guidance. Geneva, Switzerland: World Health Organization, 2022.

[9] Arriola CS, Soto G, Westercamp M, Effectiveness of whole-virus COVID-19 vaccine among healthcare personnel, Lima, Peru. Emerg Infect Dis 2022; 28:S238–s243. 36502444 10.3201/eid2813.212477 PMC9745240

[10] Centers for Disease Control Prevention. Interim guidelines for collecting, handling, and testing clinical specimens for COVID-19 Testing. 2024. Available at: https://www.cdc.gov/covid/hcp/clinical-care/clinical-specimen-guidelines.html. Accessed 19 May 2024.

[11] Wesley MG, Tinoco Y, Patel A, Performance of symptom-based case definitions to identify influenza virus infection among pregnant women in middle-income countries: findings from the Pregnancy and Influenza Multinational Epidemiologic (PRIME) study. Clin Infect Dis 2020; 73:e4321–e4328. 10.1093/cid/ciaa1697 PMC10563868 33173947

[12] Jiang L, Lee VJ, Lim WY, Performance of case definitions for influenza surveillance. Euro Surveill 2015; 20:21145. 26062645 10.2807/1560-7917.es2015.20.22.21145

[13] Davis W, Duque J, Huang QS, Sensitivity and specificity of surveillance case definitions in detection of influenza and respiratory syncytial virus among hospitalized patients, New Zealand, 2012–2016. J Infect 2022; 84:216–26. 34953903 10.1016/j.jinf.2021.12.012 PMC11753188

[14] Gupta V, Dawood FS, Rai SK, Validity of clinical case definitions for influenza surveillance among hospitalized patients: results from a rural community in north India. Influenza Other Respir Viruses 2013; 7:321–9. 22804843 10.1111/j.1750-2659.2012.00401.x PMC5779832

[15] Gill PJ, Kaziev CL, Mtaweh H, Performance of the World Health Organization (WHO) severe acute respiratory infection (SARI) case definitions in hospitalized children and youth: cross-sectional study. Lancet Reg Health Am 2025; 44:101034. 40083965 10.1016/j.lana.2025.101034 PMC11904563

[16] Rowlinson E, Peters L, Mansour A, Comparison of common acute respiratory infection case definitions for identification of hospitalized influenza cases at a population-based surveillance site in Egypt. PLoS One 2021; 16:e0248563. 33765010 10.1371/journal.pone.0248563 PMC7993808

[17] Hirve S, Chadha M, Lele P, Performance of case definitions used for influenza surveillance among hospitalized patients in a rural area of India. Bull World Health Organ 2012; 90:804–12. 23226892 10.2471/BLT.12.108837 PMC3506409

[18] Pott H, J LeBlanc J, S ElSherif M, Clinical features and outcomes of influenza and RSV coinfections: a report from Canadian immunization research network serious outcomes surveillance network. BMC Infect Dis 2024; 24:147. 38291361 10.1186/s12879-024-09033-5 PMC10826021

[19] Barry MA, Arinal F, Talla C, Performance of case definitions and clinical predictors for influenza surveillance among patients followed in a rural cohort in Senegal. BMC Infect Dis 2021; 21:31. 33413174 10.1186/s12879-020-05724-x PMC7790019

[20] Budd A, Blanton L, Grohskopf L, Chapter 6: Influenza. In: Manual for the surveillance of vaccine-preventable diseases. Centers for Disease Control and Prevention, 2017.

[21] Yang J-H, Huang P-Y, Shie S-S, Predictive symptoms and signs of laboratory-confirmed influenza: a prospective surveillance study of two metropolitan areas in Taiwan. Medicine (Baltimore) 2015; 94:e1952. 26554802 10.1097/MD.0000000000001952 PMC4915903

[22] Li J-H, Wu C-C, Tseng Y-J, Applying symptom dynamics to accurately predict influenza virus infection: an international multicenter influenza-like illness surveillance study. Influenza Other Respir Viruses 2023; 17:e13081. 36480419 10.1111/irv.13081 PMC9835452

[23] Bird O, Galiza EP, Baxter DN, The predictive role of symptoms in COVID-19 diagnostic models: a longitudinal insight. Epidemiol Infect 2024; 152:e37. 38250791 10.1017/S0950268824000037 PMC10945957

[24] Geismar C, Nguyen V, Fragaszy E, Symptom profiles of community cases infected by influenza, RSV, rhinovirus, seasonal coronavirus, and SARS-CoV-2 variants of concern. Sci Rep 2023; 13:12511. 37532756 10.1038/s41598-023-38869-1 PMC10397315

[25] World Health Organization. Global epidemiological surveillance standards for influenza. Geneva, Switzerland: World Health Organization, 2013.

[26] Prasad N, Newbern EC, Trenholme AA, Respiratory syncytial virus hospitalisations among young children: a data linkage study. Epidemiol Infect 2019; 147:e246. 31364578 10.1017/S0950268819001377 PMC6805750

[27] Centers for Disease Control and Prevention. Glossary of Influenza (Flu) Terms. 2024. Available at: https://www.cdc.gov/flu/glossary/?CDC_AAref_Val=https://www.cdc.gov/flu/about/glossary.htm. Accessed 19 May 2025.

[28] Centers for Disease Control and Prevention. Coronavirus disease 2019 (COVID-19) 2021 case definition. Available at: https://ndc.services.cdc.gov/case-definitions/coronavirus-disease-2019-2021/. Accessed 8 May 2025.

[29] World Health Organization. WHO COVID-19: case definitions: updated in public health surveillance for COVID-19. Geneva, Switzerland: World Health Organization, 2020.

[30] World Health Organization. Infection prevention and control of epidemic-and pandemic-prone acute respiratory infections in health care. Geneva, Switzerland: World Health Organization, 2014. 24983124

[31] United Kingdom Health Security Agency. Investigation and management of outbreaks of suspected acute viral respiratory infection in schools: guidance for health protection teams. 2022. Available at: https://www.gov.uk/government/publications/influenza-like-illness-ili-managing-outbreaks-in-schools/investigation-and-management-of-outbreaks-of-suspected-acute-viral-respiratory-infection-in-schools-guidance-for-health-protection-teams. Accessed 19 May 2025.

[32] United Kingdom Health Security Agency. COVID-19: epidemiology, virology and clinical features. Available at: https://www.gov.uk/government/publications/wuhan-novel-coronavirus-background-information/wuhan-novel-coronavirus-epidemiology-virology-and-clinical-features. Accessed 10 May 2025.

